# Discovery of Dual-Action Membrane-Anchored Modulators of Incretin Receptors

**DOI:** 10.1371/journal.pone.0024693

**Published:** 2011-09-14

**Authors:** Jean-Philippe Fortin, Daniel Chinnapen, Martin Beinborn, Wayne Lencer, Alan S. Kopin

**Affiliations:** 1 Molecular Pharmacology Research Center, Molecular Cardiology Research Institute, Tufts Medical Center, Tufts University School of Medicine, Boston, Massachusetts, United States of America; 2 Gastrointestinal Cell Biology and Harvard Digestive Diseases Center, Children's Hospital and Harvard Medical School, Boston, Massachusetts, United States of America; Federal University of São Paulo, United States of America

## Abstract

**Background:**

The glucose-dependent insulinotropic polypeptide (GIP) and the glucagon-like peptide-1 (GLP-1) receptors are considered complementary therapeutic targets for type 2 diabetes. Using recombinant membrane-tethered ligand (MTL) technology, the present study focused on defining optimized modulators of these receptors, as well as exploring how local anchoring influences soluble peptide function.

**Methodology/Principal Findings:**

Serial substitution of residue 7 in membrane-tethered GIP (tGIP) led to a wide range of activities at the GIP receptor, with [G^7^]tGIP showing enhanced efficacy compared to the wild type construct. In contrast, introduction of G^7^ into the related ligands, tGLP-1 and tethered exendin-4 (tEXE4), did not affect signaling at the cognate GLP-1 receptor. Both soluble and tethered GIP and GLP-1 were selective activators of their respective receptors. Although soluble EXE4 is highly selective for the GLP-1 receptor, unexpectedly, tethered EXE4 was found to be a potent activator of both the GLP-1 and GIP receptors. Diverging from the pharmacological properties of soluble and tethered GIP, the newly identified GIP-R agonists, (i.e. [G^7^]tGIP and tEXE4) failed to trigger cognate receptor endocytosis. In an attempt to recapitulate the dual agonism observed with tEXE4, we conjugated soluble EXE4 to a lipid moiety. Not only did this soluble peptide activate both the GLP-1 and GIP receptors but, when added to receptor expressing cells, the activity persists despite serial washes.

**Conclusions:**

These findings suggest that conversion of a recombinant MTL to a soluble membrane anchored equivalent offers a means to prolong ligand function, as well as to design agonists that can simultaneously act on more than one therapeutic target.

## Introduction

Glucose-dependent insulinotropic polypeptide (GIP) and glucagon-like peptide 1 (GLP-1) are structurally related incretin hormones that are released from intestinal enteroendocrine cells in response to food intake. Both hormones share important physiological roles, notably in maintaining blood glucose homeostasis by potentiating glucose-stimulated insulin secretion from pancreatic β-cells. GLP-1 and GIP also promote the expansion of pancreatic islet mass via induction of β-cell proliferation and survival [1], [2].

The GLP-1 receptor (GLP-1R) is a well-established therapeutic target for the treatment of type 2 diabetes (T2D) [3]. In addition to enhancing insulin secretion from pancreatic beta cells, stimulation of this receptor also reduces blood glucose levels via effects on extrapancreatic tissues including the gastrointestinal tract and the brain [1]. GLP-1 triggers delayed gastric emptying which in turn slows nutrient absorption thus attenuating the rise in blood glucose levels. In the central nervous system, GLP-1 has been shown to inhibit feeding behavior and to promote weight loss by stimulation of cognate receptors, thereby further contributing to improved glucose tolerance [1], [4].

Understanding the multifunctional role of GLP-1 in modulating glucose homeostasis led to interest in developing mimetics of this peptide as drugs for the treatment of T2D. The lizard peptide exendin-4 (Exenatide), a potent agonist of the GLP-1 receptor, was the first incretin mimetic to be marketed as a treatment for T2D [5]. A more recent addition to the therapeutic armamentarium is liraglutide, a stable long-acting GLP-1 derivative [6]. As complementary therapeutics, inhibitors of dipeptidyl dipeptidase-4, the endogenous enzyme that rapidly degrades GLP-1, have also been introduced into the clinic [5].

With respect to GIP, previous studies support that selected mimetics exhibit potent antidiabetic actions in animal models of T2D, resulting in improved glucose tolerance, insulin secretion and β-cell survival [1], [7]. Prior concerns regarding a partial loss of GIP-R responsiveness in patients with T2D have been tempered by more recent studies suggesting that this defect may be reversible once blood glucose levels are reduced (e.g., by treatment with other drugs) [5], [8]. In light of these insights, there has been a renewed interest in developing GIP-R agonists, as well as dual incretin receptor activators for T2D [9], [10].

Both the GIP receptor (GIP-R) and the GLP-1R belong to the glucagon subfamily of class B1 G protein-coupled receptors (GPCRs). Although pharmacologically distinct and highly selective for corresponding peptides, these two incretin receptors both trigger Gs-mediated cAMP production in response to agonist stimulation. Structure-function analyses and recent crystallographic studies support a two-domain model for incretin recognition and receptor activation [11], [12], [13]. As proposed for most class B1 GPCRs, it is postulated that the C-terminal α-helical portion of either GLP-1 or GIP initially binds the N-terminal extracellular domain of cognate receptors; this interaction in part defines both ligand affinity and specificity. As a second step, the N-terminal segment of the hormone interacts with the receptor transmembrane domains and connecting extracellular loops. This, in turn, leads to conformational changes in the receptor protein that trigger intracellular signal transduction [13].

We recently reported the development of membrane-tethered ligands (MTLs) as probes to investigate the function of class B1 GPCRs both *in-vitro* and *in-vivo* [14], [15]. These recombinant constructs are designed to encode a peptide hormone, an epitope tag and a membrane-anchoring sequence (transmembrane domain or glycosylphosphatidylinositol moiety), all coupled by intervening flexible protein linkers [14], [15]. As with soluble peptides, the N-terminal amino acids of both GIP and GLP-1 MTLs include critical activity determinants; substitution within this domain can lead to increased or decreased activity [15]. Using the membrane-tethered ligand technology, the present study aimed to optimize modulators of incretin receptors, as well as to better define how local plasma membrane-anchoring may influence ligand function. We report the discovery of both recombinant membrane-tethered ligands and lipidated soluble peptides that display dual incretin receptor agonism. Our results demonstrate the utility of membrane-tethered ligands in optimizing peptide function, as well as the feasibility of mimicking the properties of such constructs with soluble ligands that are chemically coupled to a lipidic membrane anchor.

## Results

### Selected amino acid substitutions targeting position 7 of tethered GIP enhance efficacy at the GIP-R

**Figure 1:**
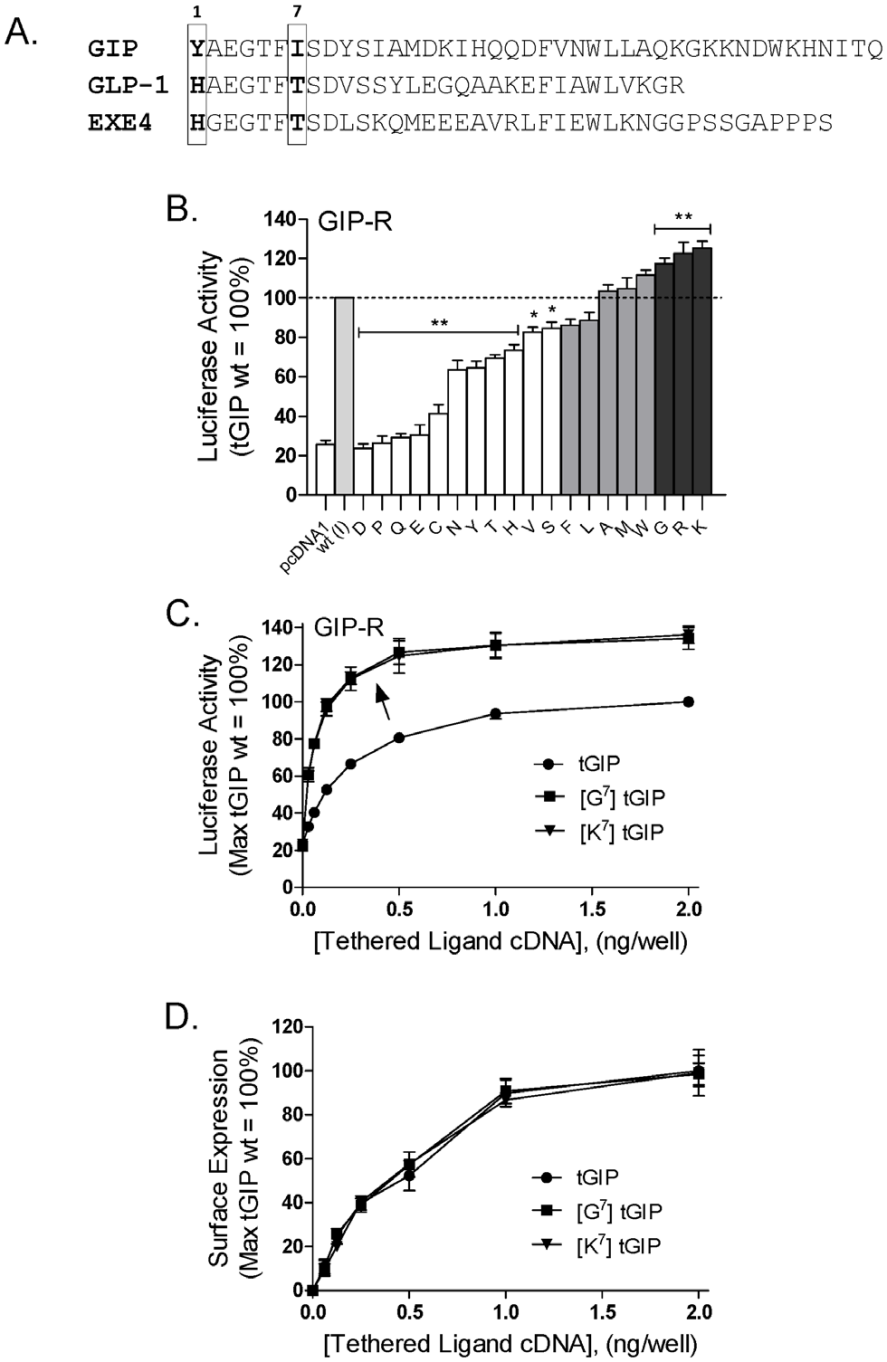
Functional consequences of amino acid substitutions targeting position 7 in tethered GIP. HEK 293 cells were transiently transfected with the GIP-R cDNA and a tethered GIP construct, together with a CRE_6X_-LUC reporter gene construct. Twenty four hours post-transfection, luciferase activity was quantified as described in Methods. (A) Sequence comparison of human GIP and GLP-1 hormones, and of EXE4. Position 1 represents the N-terminal residue of the peptides. Within the first 9 amino acids, GIP and GLP-1 differ only at positions 1 and 7 (boxes). (B) Serial substitution of isoleucine 7 in tGIP by each of the other 19 amino acids leads to a wide range of tethered ligand-induced activities at the GIP-R. The constructs showing activities similar to wild-type are shown in light gray. The constructs displaying significantly reduced or increased activity versus wild-type tGIP are shown in white and dark gray, respectively. (C) Assessment of tethered ligand activity with increasing amounts of transfected cDNA, confirm the enhanced activity of the [G^7^] tGIP and [K^7^] tGIP derivatives (arrow), relative to wild-type tGIP. (D) Comparable cell surface expression of tGIP, [G^7^] tGIP and [K^7^] tGIP, as assessed by ELISA following transfection of increasing amounts of corresponding cDNAs. All activity or expression data were normalized relative to the wild-type tethered GIP values. Data represent the mean ± SEM from at least 4 independent experiments, each performed at least in quadruplicate. The activity of mutant versus wild-type tGIP were compared by analysis of variance followed by Dunnett's post-test; significance, *, *p*<0.05; **, *p*<0.01.

We have previously established that position 7 (Figure 1A) represents an important efficacy and selectivity determinant of membrane-tethered incretins [15]. We reported that substitution of isoleucine 7 in tGIP with the corresponding GLP-1 amino acid, threonine 7 (in the context of a second tGIP substitution Y^1^→H^1^) was sufficient to convert tGIP to a GLP-1R agonist [15]. Notably, position 7 among class B1 hormones has also been proposed to be the central element of a putative helix N-capping motif important for hormone activity and stability [16], [17].

To further explore the role of this position as an activity determinant of tGIP, this residue was serially replaced by each of the other 19 naturally-occurring amino acids. Ligand-induced function at the GIP-R was then assessed utilizing a CRE luciferase reporter gene assay [15]. This screen supports the importance of position 7 as an efficacy determinant of tGIP, and led to the identification of constructs with markedly altered GIPR-mediated activity, relative to unmodified tGIP (Figure 1B). Substitution with the negatively charged amino acids aspartic acid (D) and glutamic acid (E) markedly impaired activity, while introduction of the positively charged lysine (K) and arginine (R) residues led to a gain-of-function. Interestingly, substitution of residue 7 with glycine (G), which has a minimal side chain, also significantly increased ligand function. In contrast, non-polar residues including alanine (A), leucine (L), methionine (M), when introduced at position 7, had no apparent impact on ligand activity.

To better understand the mechanism underlying increased tethered ligand function, the K and G substituted derivatives were selected for further analysis. Enhanced function of these tethered ligands was confirmed over a wide range of cDNA concentrations (Figure 1C). Both of these tethered gain-of function analogs were selective for the GIP-R, displaying no activity at the GLP-1R (data not shown; Table 1). To evaluate whether the increase in activity of [G^7^]tGIP and [K^7^]tGIP was the result of higher tethered ligand expression, surface levels of corresponding constructs were quantified (Figure 1D). ELISA data exclude that enhanced activity is the consequence of increased tethered ligand expression.

**Table 1:** Relative activity of wild-type and G^7^/K^7^ tethered incretin derivatives at the GIP and GLP-1 receptors.

	GIP-R	GLP-1R
tGIP	++	−
[G^7^]tGIP	+++	−
[K^7^]tGIP	+++	−
tGLP-1	−	++
[G^7^]tGLP-1	−	++
[K^7^]tGLP-1	−	−
tEXE4	+++	++
[G^7^]tEXE4	+	++
[K^7^]tEXE4	+	−

Notably, tethered EXE4 is a potent activator of both receptors.

### Impact of the G^7^ and K^7^ substitutions on the function of the related tGLP-1 and tEXE4 constructs

**Figure 2:**
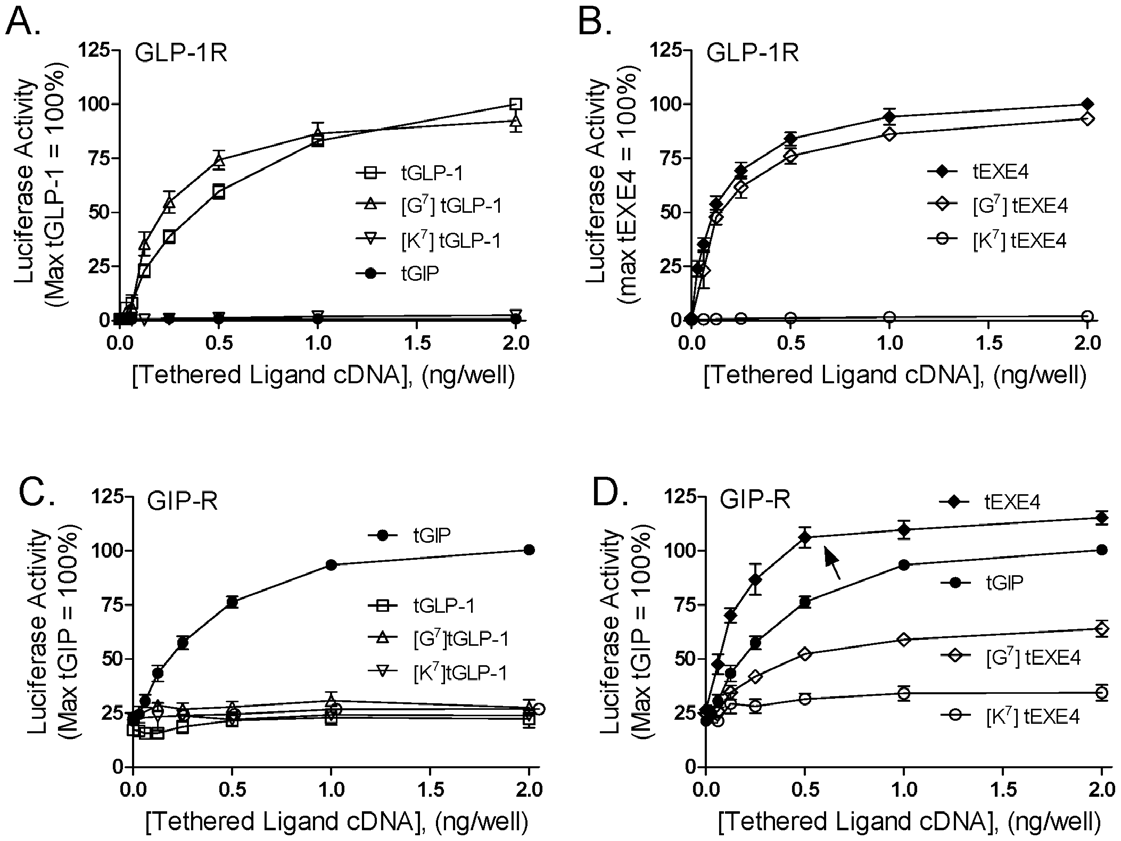
Activity and selectivity of wild-type and G^7^/K^7^ derivatives of tGLP-1 and tEXE4. Activity of tethered GLP-1 and tethered EXE4 constructs at both the GLP-1R (A and B) and the GIP-R (C and D) are compared. HEK 293 cells were transiently transfected with cDNAs encoding the GLP-1R or GIP-R, a tethered ligand, and a CRE_6X_-LUC reporter gene construct. Twenty four hours post-transfection, ligand-induced activity was quantified as described in Methods. All activity data were normalized relative to the corresponding wild-type tethered GIP, GLP-1 or EXE4 construct, as indicated. Unexpectedly, a tethered version of wild-type EXE4 displayed high activity not only at the GLP-1R, but also at the GIP-R (arrow). Data represent the mean ± SEM from at least 4 independent experiments, each performed at least in quadruplicate.

The N-terminus of GIP in the vicinity of residue 7 is highly homologous to that of the GLP-1R agonists GLP-1 and EXE4 (Figure 1A). We therefore investigated whether G^7^ and K^7^ substitutions would also enhance the function of the latter peptides on their target receptor. As demonstrated previously, wild-type forms of tethered GLP-1 and EXE4 were potent activators of the GLP-1R [15]. Substitution of position 7 with glycine ([G^7^]tGLP-1 and [G^7^]tEXE4) resulted in activity comparable to the corresponding unmodified ligands when assessed at the GLP-1R. In contrast, introduction of the K^7^ substitution in tGLP-1 and tEXE4 completely abrogated the ability of both ligands to trigger GLP-1R mediated signaling (Figure 2A–B). As observed with tGIP, position 7 in tGLP-1 and tEXE4 was thus critical for efficacy yet in the latter peptides neither substitution enhanced activity at the cognate GLP-1R.

We previously demonstrated that the residue in position 7 of related incretin ligands can determine the selectivity of tethered ligands for the GLP-1R versus the GIP-R. On this basis, we next evaluated the function of tGLP-1 and tEXE4-based constructs at the GIP-R. Wild-type as well as both the G^7^ and K^7^ tGLP-1 analogs were inactive at the GIP-R (Figure 2C). Unexpectedly, however, the wild-type EXE4 membrane-tethered construct was a potent GIP-R activator, in fact triggering activity levels higher than tGIP itself. Both [G^7^]tEXE4 and [K^7^]tEXE4 analogs displayed reduced GIP-R mediated signaling, relative to unmodified tGIP and tEXE4 (Figure 2D).

Taken together, our studies have identified two tethered ligands, [G^7^]tGIP and tEXE4, which each exhibit GIP-R mediated signaling higher than that of tGIP. Notably, tEXE4 was a dual agonist with full activity at both the GLP-1R and GIP-R. A summary of the activity and selectivity of wild-type, as well as G^7^ or K^7^ substituted tethered incretin constructs is shown in Table 1.

### Soluble [G^7^]GIP and EXE4 are low potency GIP-R agonists

**Figure 3:**
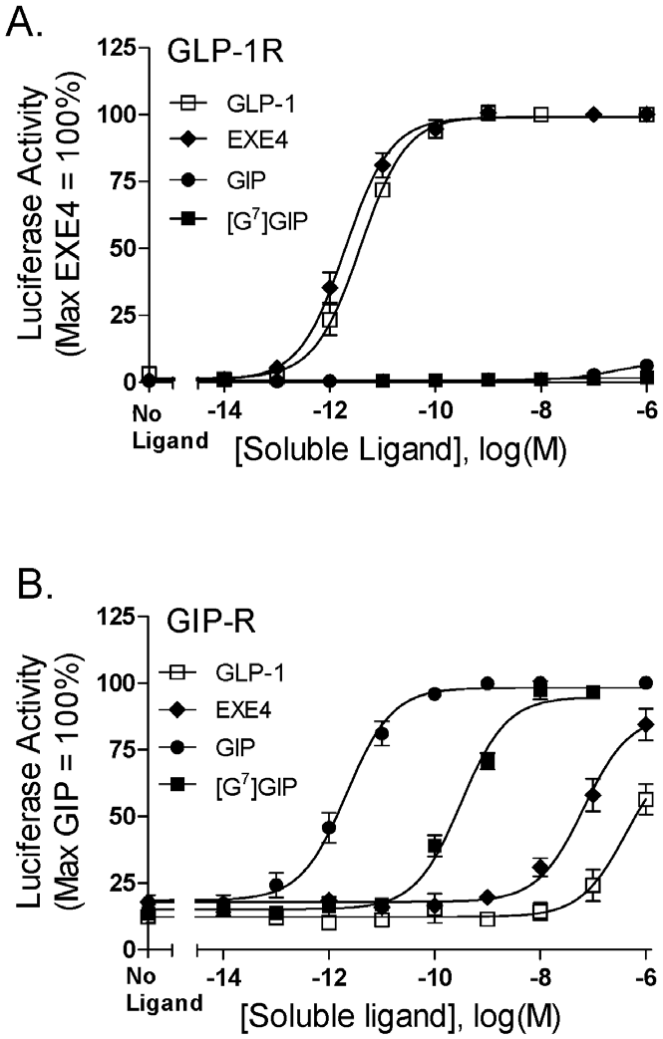
Signaling potency of soluble incretin peptides at the GLP-1 and GIP receptors. Soluble ligand concentration-response curves for GLP-1, EXE4, GIP and [G^7^] tGIP were assessed on cells expressing the GLP-1R (A) or the GIP-R (B). HEK293 cells were transiently transfected with cDNAs encoding the indicated receptor and a CRE_6X_-LUC reporter gene construct. Twenty four hours post-transfection, cells were stimulated for 6 hours with soluble peptides; ligand-induced activity was then quantified. All activity data were normalized relative to soluble EXE4 (A) or GIP (B). Both soluble GLP-1 and EXE4 were highly potent agonists at the GLP-1R, and low potency agonists at the GIP-R. GIP and [G^7^] tGIP only showed activity at the GIP-R. Data represent the mean ± SEM from at least 4 independent experiments, each performed at least in quadruplicate.

To better define the impact of membrane-tethering on ligand function, comparative studies were done to examine the effects of soluble EXE4, GLP-1, GIP and [G^7^]GIP at the GIP-R and GLP-1R. Soluble EXE4 and GLP-1 were full agonists at the GLP-1R, whereas GIP and [G^7^]GIP had minimal if any activity (Figure 3A). On cells expressing the GIP-R, [G^7^]GIP and EXE4 were agonists with potencies ∼100-fold and ∼50,000 fold lower than unmodified GIP, respectively (Figure 3B). GLP-1 was an even lower potency agonist. These observations contrast with the gain-of-function observed at the GIP-R when tethered constructs, [G^7^]tGIP and tEXE4, were compared to tGIP wild-type (Figure 1C and 2D).

### Effects of tethered agonists on cell surface expression of the GIP-R

To investigate the mechanism underlying the enhanced function of [G^7^]tGIP and tEXE4 (vs tGIP), we examined how tethered ligands modulate GIP-R surface expression using a previously described ELISA assay [18]. The effects of soluble ligands were assessed in parallel.

**Figure 4:**
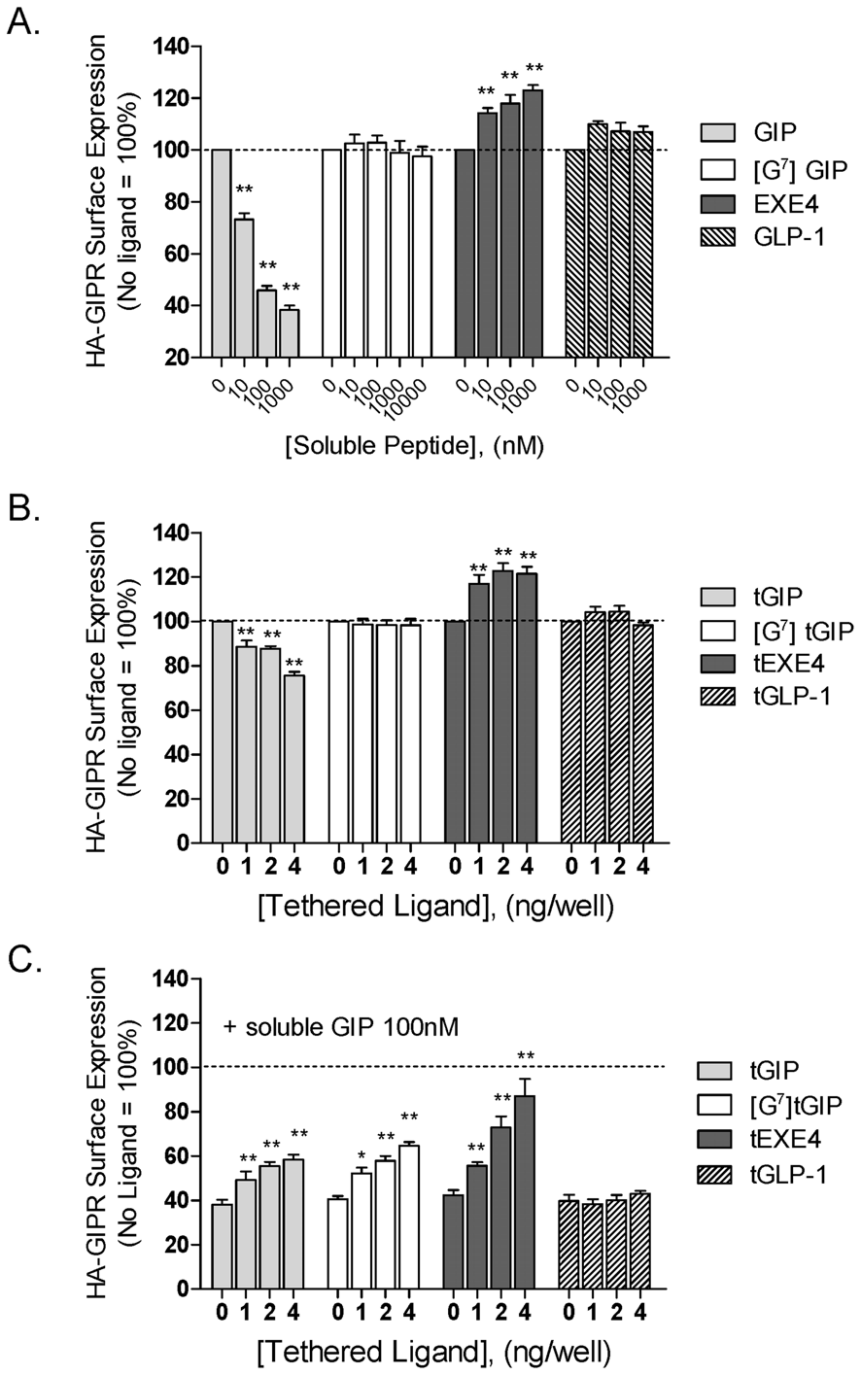
Comparison of soluble versus tethered ligand-induced modulation of GIP-R surface expression. The impact of soluble peptide (A), tethered ligands (B) and the interaction between soluble and tethered ligands on HA-GIPR surface expression (C) was measured by ELISA. HEK 293 cells were transfected with a plasmid encoding the HA-tagged GIP-R, with or without increasing amounts of a tethered ligand cDNA. Twenty four hours after transfection, soluble ligands were added in selected wells, as indicated. In each experiment, receptor expression was evaluated 48 hours after transfection. Soluble and tethered GIP were the only ligands able to down-modulate HA-GIPR expression (A, B). Tethered GIP, [G^7^] tGIP and tEXE4 interfered with soluble GIP-induced endocytosis (C). Data represent the mean ± SEM from at least 4 independent experiments, each performed in 12 replicates. The expression of HA-GIPR in the absence vs presence of soluble or tethered ligand were compared by analysis of variance followed by Dunnett's post-test; significance, *, *p*<0.05; **, *p*<0.01.

As anticipated based on the literature [19], surface expression of the HA-tagged GIP-R was markedly reduced after prolonged incubation with increasing doses of soluble GIP (Figure 4A). In contrast, high concentrations of the soluble agonists [G^7^]GIP or EXE4 failed to down-regulate receptor levels. In fact, overnight incubation with EXE4 significantly increased receptor levels. GLP-1, which is inactive at the GIP-R, also showed no effect on GIP-R membrane expression levels (negative control).

As observed with soluble GIP, expression of the wild-type tethered GIP construct significantly decreased GIP-R surface levels (Figure 4B). In contrast, despite being highly active in triggering signaling, [G^7^]tGIP had no significant effect on GIP-R expression (Figure 4B). As observed with the corresponding soluble ligand, tEXE4 significantly increased GIP-R levels. The inactive tGLP-1 construct showed no effect on GIP-R membrane expression levels.

We further explored how tethered ligands influence putative GIP-induced endocytosis (Figure 4C). Increased expression of tGIP, [G^7^]tGIP and tEXE4 each significantly attenuated receptor endocytosis induced by soluble GIP (100 nM, 18 h). In contrast, tGLP-1 had no impact on GIP-R endocytosis (negative control). Taken together, these data support that selected agonists (either soluble or tethered) can activate the GIP-R without triggering endocytosis.

### Impact of tethered activators on the subcellular distribution of the GIP-R

The impact of soluble GIP versus selected tethered activators on the subcellular localization of the GIP-R was further characterized by confocal imaging. For this purpose, we generated a fusion protein which includes the GIPR tagged with a monomeric cherry fluorescent protein (CHE) on the C-terminus (GIPR-CHE). Fluorescent tethered ligands were also generated with the green fluorescent protein (GFP) at the C-terminal end of the constructs (Figure S1A–B). Receptor and tethered ligand fusion proteins each showed activity similar to their corresponding untagged counterparts when assessed using LUC-reporter gene assay (data not shown).

**Figure 5:**
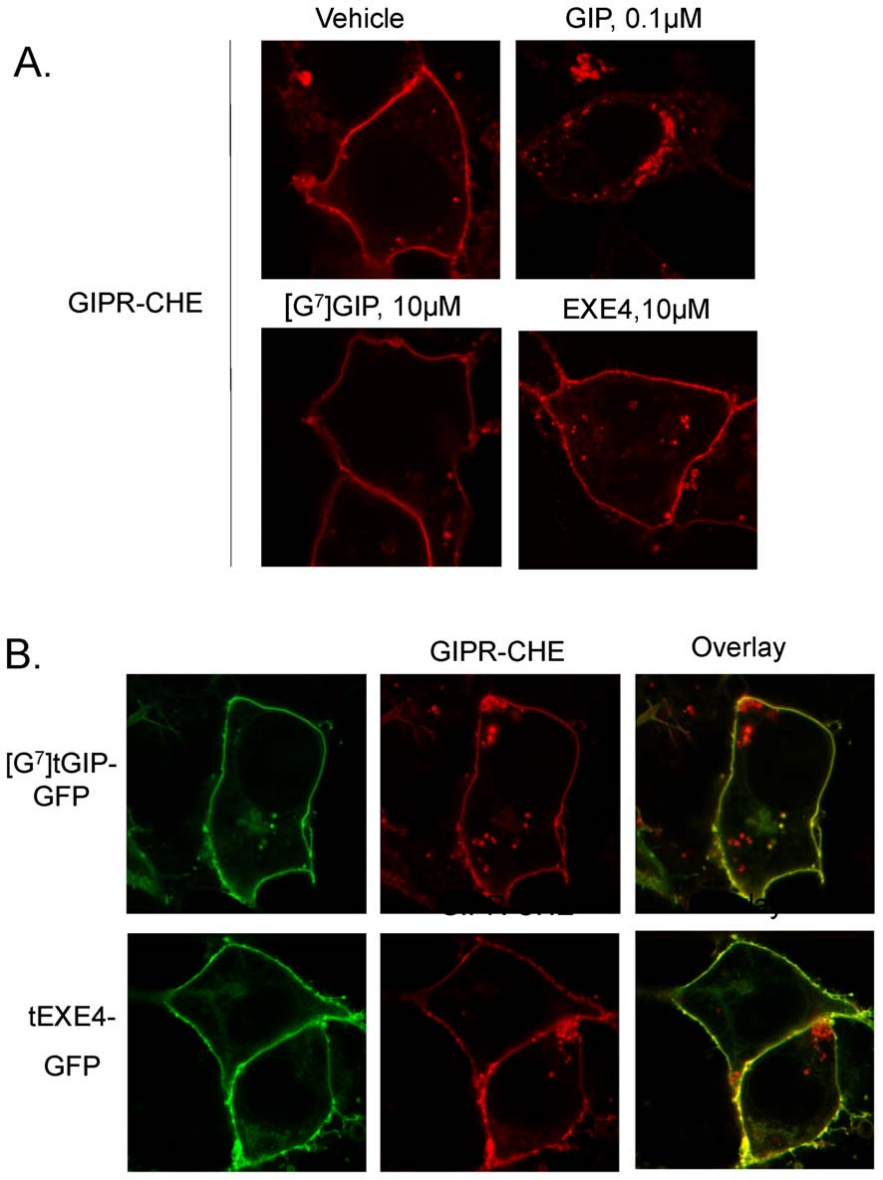
Analysis of soluble versus tethered ligand-induced GIP-R endocytosis using fluorescently-tagged constructs. The impact of selected soluble peptides (A) and tethered ligands (B) on the subcellular distribution of GIPR-CHE was studied using confocal microscopy. HEK 293 cells were transiently transfected with a plasmid encoding the GIPR-CHE, with or without cDNA encoding a GFP-tagged tethered ligand. Twenty-four hours later, the cells were treated for 18 hours with a soluble peptide or the corresponding vehicle, as indicated. The subcellular distribution of receptor was then visualized. Whereas soluble GIP triggered massive endocytosis, other soluble and tethered ligands had no apparent effect.

Under basal conditions, GIPR-CHE expressing cells showed plasma membrane-labeling, as well as occasional punctate intracellular staining. Prolonged treatment with soluble GIP triggered massive GIPR-CHE endocytosis, characterized by fluorescence accumulation in intracellular compartments (Figure 5A). The subcellular localization of internalized GIP-Rs was investigated using a Rab7-GFP marker which labels intracellular endosomal compartments [20] (Figure S2). Supporting that chronic treatment with GIP leads to GIPR-CHE translocation to late endosomes, both Rab7-GFP and the receptor colocalized intracellularly following agonist treatment.

**Figure 6:**
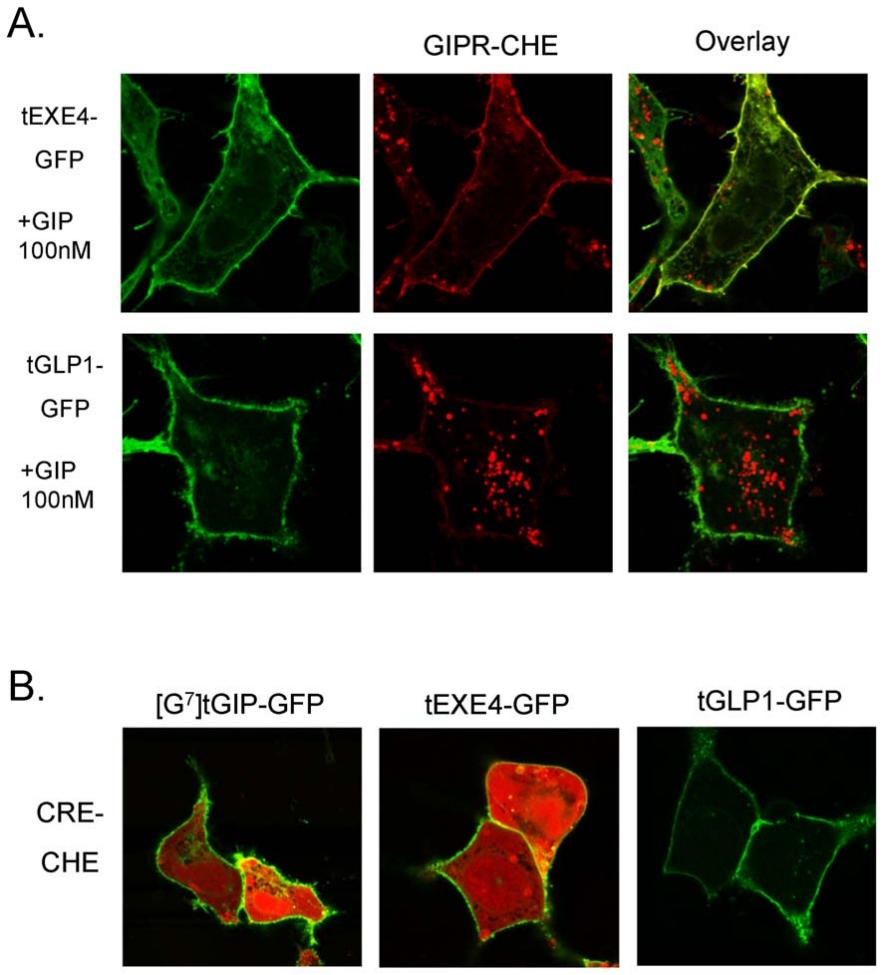
Impact of tethered ligand expression on soluble GIP-induced endocytosis of the GIP-R. (A) Expression of tEXE4-GFP blocked soluble GIP induced-endocytosis of GIPR-CHE. Cells were transfected with plasmids encoding GIPR-CHE and tEXE4-GFP or tGLP1-GFP. Twenty-four hours later, the cells were treated for 18 h with media containing 100 nM of soluble GIP, followed by confocal microscopy imaging. (B) Both [G^7^]tGIP-GFP and tEXE4-GFP induced high receptor-dependent signaling, relative to the inactive tGLP1-GFP construct. HEK293 cells were transfected with plasmids encoding the untagged GIP-R, a GFP-tagged tethered ligand, together with a CRE_6x_-CHE reporter construct, as described in *Methods*. Intracellular CHE accumulation was visualized 48 hours after transfection.

Consistent with our ELISA results, the cell surface distribution of GIPR-CHE was not altered following treatment with high doses of either soluble [G^7^]GIP or EXE4 (Figure 5A) . Further supporting that corresponding [G^7^]tGIP-GFP and tEXE4-GFP membrane-tethered ligands do not induce receptor endocytosis, both constructs were colocalized with the GIPR-CHE at the plasma membrane (Figure 5B). Furthermore, tEXE4-GFP blocked receptor endocytosis induced by soluble GIP, whereas tGLP1-GFP had no effect (Figure 6A).

To visually confirm GIPR-mediated signaling in cells expressing GFP-tagged tethered activators, a CRE_6x_-CHE reporter gene was generated (Material and methods, Figure S1). Consistent with luciferase assays, both [G^7^]tGIP-GFP and tEXE4-GFP triggered GIP-R dependent signaling (intracellular accumulation of CHE) (Figure 6B). In contrast, the surface-expressed tGLP1-GFP construct (negative control) failed to activate the GIP-R. These data further support that selected tethered agonists may trigger signaling without inducing receptor internalization.

### Lipidated peptides can recapitulate selected properties of recombinant tethered ligands

**Figure 7:**
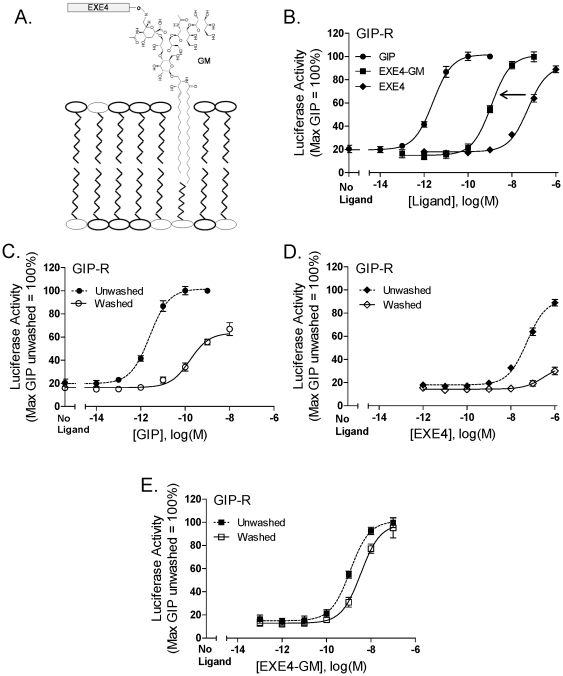
Signaling properties of a ganglioside-EXE4 conjugate. (A) Cartoon of an EXE4-ganglioside conjugate (EXE4-GM) interacting with the cellular membrane. (B) Potency of EXE4-GM compared with that of soluble EXE4 and GIP at the GIP-R. (C–E) Unmodified GIP (C) and EXE4 (D) showed reduced function with serial washes, whereas the activity of EXE4-GM (E) persisted. HEK293 cells were transiently transfected with a cDNA encoding the GIP-R, together with a CRE_6X_-LUC reporter gene construct. Twenty four hours post-transfection, cells were stimulated for 15 minutes with increasing concentrations of the indicated ligands. As indicated, selected wells were then washed three times with serum-free media, and plates were further incubated for an additional 4 hour period. Luciferase activity was subsequently measured as described in *Methods*. Activity data were normalized relative to the soluble GIP-induced maximum. Data represent the mean ± SEM from at least 4 independent experiments, each performed at least in quadruplicate.

Based on the enhanced potency of EXE4 at the GIP-R following membrane-tethering (Figure 2D), we postulated that similar effects might be reproduced using a soluble membrane-anchored version of this peptide. To explore this possibility, an EXE4 peptide linked to a GM1 ganglioside (acting as the lipidic membrane-anchoring moiety) was characterized (EXE4-GM; Figure 7A). Supporting that GM1 could act as an effective membrane anchor, previous work demonstrated rapid plasma membrane insertion of fluorescent gangliosides when added to cultured cells [21].

As predicted by recombinant membrane-tethered ligand, the signaling potency of the EXE4-GM compound was enhanced by ∼50 fold relative to the EXE4 peptide when assessed at the GIP-R (Figure 7B; pEC_50_ = 7.28±0.25 versus 8.94±0.05 for EXE4 and EXE4-GM, respectively; mean± SEM). When measured at its primary target, the GLP-1R, EXE4-GM remained a high potency agonist (EC_50_ = 48 pM, pEC_50_ = 10.25±0.10), whereas this construct lacked activity in cells transfected with the empty vector pcDNA1 in place of receptor cDNA (data not shown).

To further test the hypothesis that increased potency of EXE4-GM at the GIP-R is attributable to local membrane-anchoring, we analyzed the persistence of agonist-induced signaling with serial washes [22]. Whereas potency and efficacy of GIP and unmodified EXE4 were markedly decreased following washes, the activity of EXE4-GM was minimally affected (Figure 7C–E). This observation supports that lipidation of EXE4 enhances binding of the ligand to the cell surface, reminiscent of a recombinant MTL.

## Discussion

We have performed a comparative analysis of soluble versus tethered peptides targeting the GIP-R and GLP-1R that has begun to reveal how membrane-anchoring in combination with single amino acid substitutions alters the actions of corresponding ligands. We have taken advantage of these insights to generate a first generation membrane-anchored, peptide-lipid conjugate which activates both incretin receptors.

Our studies initially focused on the role of residue 7 as a critical determinant of agonist activity at the GIP-R. This position was selected for exploration based on two intersecting lines of investigation. In a prior study, we had demonstrated that interchange of GIP and GLP-1 residues in this position contributed to the activity of both tethered ligands at their cognate receptors [15]. In addition, position 7 is part of a highly conserved helix capping structure common to class B1 hormones [16], [17]. The corresponding motif is predicted to favor α-helix stability [23]. In the current study, serial substitutions in place of isoleucine 7 in tGIP led to constructs with variable levels of activity at the GIP-R. Detailed follow-up analysis revealed that both the G^7^ and K^7^ substitutions led to enhanced activity which was not attributable to an increase in construct expression and was thus of particular interest.

Supporting the role of position 7 as an efficacy/selectivity determinant of class B1 ligands at their cognate receptor, introduction of K^7^ in the related tGLP-1 and tEXE4 peptides markedly reduced activity at the GLP-1R. In contrast, the G^7^ substitution was well-tolerated when introduced into tGLP-1 and tEXE4. Extending this analysis to other closely-related class B1 peptides, introduction of G^7^ into tethered forms of the vasoactive intestinal polypeptide (VIP) and the pituitary adenylate cyclase-activating peptide (PACAP27), markedly potentiated ligand activity at the VPAC-1R (Figure S3). Previous structure-function studies on soluble PACAP complement our findings and support a role of this capping structure for both peptide activity and stability [24], [25]. A recent study further reported that introduction of A^7^ in PACAP38 leads to a superagonist peptide [26]. Taken together, our results and the literature suggest the importance of position 7 as an efficacy/selectivity determinant in both soluble and tethered forms of hormones acting on class B1 GPCRs.

In the current investigation, we have also shown that membrane-tethered EXE4 displays an unexpected high activity level at the GIP-R, in fact exceeding that of tethered GIP. Earlier studies have primarily focused on EXE4 as a GLP-1R agonist [1]. It is of note that membrane-tethering failed to potentiate the activity of GLP-1, despite the fact that this low potency GIP-R agonist shares a similar N-terminal sequence with EXE4. Moreover, rendering the N-terminal tGLP-1 domain identical to tEXE4 using a G^2^ substitution did not further increase GIP-R-dependent activity (data not shown). These results suggest that unique determinants of EXE4 underlie its enhanced ability to effectively modulate the GIP-R as a tethered ligand. Among other explanations, this difference could in part stem from the fact that GLP-1 has a less stable secondary helical structure, when compared with EXE4 [12].

To further explore how tethering influences ligand activity/potency, we compared the properties of soluble versus tethered forms of GIP, [G^7^]GIP and EXE4. In contrast to the enhanced function of tethered EXE4 (relative to tethered GIP), the soluble EXE4 peptide exhibits a ∼5,000 fold lower potency than soluble GIP. Similarly, a [G^7^]GIP derivative had lower potency than GIP when assessed as a soluble peptide. Such differences between the relative activities of soluble ligands and tethered constructs support our previous suggestion that membrane-anchoring creates a high local concentration of ligand in the receptor vicinity, and thus potentiates the ability of selected low potency agonists to activate a receptor [15].

To better understand the enhanced activity of [G^7^]tGIP and tEXE4, we studied the ability of these ligands to modulate GIP-R cell surface expression levels. Consistent with a prior report [27], our ELISA and microscopy analyses demonstrate that prolonged treatment with soluble GIP triggers receptor internalization and trafficking to the endosomal compartment. Similarly, expression of the tethered version of GIP also lowers surface expression of the GIP-R. In contrast, both [G^7^]tGIP and tEXE4, while signaling to a level exceeding that of tGIP, do not decrease GIP-R surface expression after long-term stimulation. Furthermore, these constructs block receptor endocytosis induced by soluble GIP. Reminiscent of the differential effects on signaling vs internalization observed with membrane-tethered ligands, a recent study reported biased activators of opioid receptors that trigger efficacious G-protein dependent signaling, while simultaneously acting as antagonists of receptor internalization and down-regulation [28]. It is possible that the lack of ligand-induced receptor endocytosis contributes to the increased efficacy of [G^7^]tGIP and tEXE4. The proposed link between reduced internalization and enhanced signaling is supported by additional studies on both class A and B GPCRs including the GLP-1R and GIP-R. These experiments showed that interfering with the process of receptor internalization/desensitization can amplify the level of ligand-induced G-protein mediated signaling [19], [29], [30], [31].

It is of note that long-term incubation with soluble or tethered EXE4 slightly up-regulated GIP-R surface levels. As observed with other GPCRs, such increased expression could result from an inhibition of constitutive receptor endocytosis and recycling [32]. Alternatively, it is possible that recombinant membrane-tethered ligands interact with newly synthesized GIP-Rs and favor the processing of these receptors to the cell surface [33].

One of the challenges of membrane-tethered ligands if they are to be used clinically is delivery. Recombinant constructs are amenable to gene therapy; however this is accompanied by additional risks [34]. To circumvent this concern, we now report that selected properties of recombinant tethered ligands can be recapitulated, albeit in part, using a synthetic lipidated peptide. Notably, an EXE4 derivative fused with a ganglioside (acting as a membrane anchor) exhibited significantly higher potency than the free EXE4 peptide when assessed at the GIP-R. Previous work demonstrated that plasma membrane incorporation of exogenously added gangliosides is influenced by a number of parameters including the temperature, incubation time, as well as the presence of lipid carriers [21]. Optimization of delivery conditions and membrane-anchored ligand structure (e.g. ligand composition, length of the linker between the peptide and lipid group, nature of the membrane-anchor) may further enhance the potency of lipidated EXE4 derivatives.

Increasing evidence supports that the potency and duration of drug action are influenced by the extent of target association [35]. This concept has been primarily ascribed to small lipophilic molecules, for which incorporation in the lipid bilayer is postulated to affect the residency time near the receptor. A well-known example is the long-acting β_2_ adrenergic agonist salmeterol, which has a high tendency to partition into cell membranes due to an extended lipophilic side chain [36]. Interestingly, as a mechanism underlying its prolonged *in vivo* effects, studies have proposed that salmeterol may be less prone to induce β_2_ adrenergic receptor endocytosis, relative to full agonists [37]. Importantly, sustained receptor activation by salmeterol survives extensive wash procedures [22], a result that was confirmed in our hands (data not shown). Our observation that receptor-mediated signaling induced by the EXE4-GM derivative persists under similar wash conditions is consistent with membrane-insertion of the lipidated peptide. Our data further support (as documented for selected small molecules), that hindering diffusion away from target receptors may offer a strategy to potentiate the actions of peptidic ligands.

In the design of effective incretin mimetics for type 2 diabetes, recent efforts have focused on improving their pharmacokinetic properties, including plasma half-life [1]. Notably, acylated derivatives of both GLP-1 (e.g. Liraglutide) and GIP are being developed as long-acting antidiabetic drugs [6], [9]. Structurally, the design of such analogs markedly differs from EXE4-GM in that fatty-acids are covalently linked to lysine residues within the C-terminal portion of the hormones [9], [38]. Interestingly, despite the addition of large substituents within the domain proposed to bind the receptor [11], [12], many of these lipidated incretins (when pharmacologically assessed *in vitro*) have potencies similar to or even higher than those of the corresponding unmodified hormones [9], [39]. Although fatty-acid conjugation is known to prolong circulating peptide half-life by facilitating binding to plasma proteins, previous work also suggests that lipid groups increase affinity for cellular membranes [40]. Extending from our results with EXE4-GM, it is possible that interaction with the plasma membrane also impacts the properties of some recently developed acylated incretin analogs.

Therapeutics mimicking the activities of multiple gut hormones represent an emerging theme in the treatment of diabetes and obesity [4]. The promise of this approach is highlighted by recent reports of a dual peptide agonist, acting at both the GLP-1 and glucagon receptors, which reverses obesity in rodents [41], [42]. Although dual-action activators of the GLP-1R and GIP-R have yet to be reported, recent literature supports the potential of such molecules for the treatment of T2D [4], [10], [43]. Our current data suggest that a membrane-anchored form of exendin-4 may represent a promising lead molecule which with further optimization may result in a potent GIP-R and GLP-1R dual-agonist. Such ligands could theoretically combine the beneficial effects of GLP-1 on gastric emptying, appetite control and body weight, with an enhanced ability to improve β-cell function and mass via both receptors [10]. It is of note that the diminished insulinotropic actions of GIP observed in T2D patients are recovered with normalization of blood glucose levels [5], [8]. Consequently, GLP-1R mediated improvement of hyperglycaemia could gradually enhance the antidiabetic actions of a dual agonist simultaneously acting via GIP-Rs.

In conclusion, this study reports the identification and molecular characterization of novel membrane-anchored ligands displaying GIPR-selective, as well as dual incretin receptor agonism. This work further suggests that the activity of selected efficacious membrane-tethered peptides can be recapitulated by designer lipid-peptide conjugates. These optimized lipidated ligands may also provide a means to produce sustained receptor-mediated activity in targeted tissues. Considering that our recombinant approach was previously shown to accommodate the vast majority of class B1 GPCR hormones [14], [15], it may be anticipated that additional peptides belonging to this family will show altered and potentially therapeutically useful pharmacologic properties when conjugated to gangliosides or other membrane-anchors.

## Materials and Methods

### Generation of Tethered Ligand, GPCR and Reporter Gene Constructs

The pcDNA1.1 plasmids encoding membrane-tethered versions of GIP, GLP-1 and EXE4, as well as the wild-type GIP-R and GLP-1R, have been described previously [15], [18], [44]. Amino acid substitutions were introduced into selected tethered ligands using oligonucleotide-directed site-specific mutagenesis, as previously reported [45]. Tethered ligand constructs with enhanced green fluorescent protein (GFP) at the C-terminus were generated using a stepwise approach. Using site-directed mutagenesis, stop codons were substituted by a sequence encoding a linker (five glycine-serine (GS) repeats) and a *XhoI* restriction site. The GFP coding sequence (pEGFP-N3; Clontech Laboratories Inc. Palo Alto, CA) was amplified by polymerase chain reaction (PCR) using sense (5′-ACCGCTCGAGATGGTGAGCAAGGGCGAGGAG-3′) and antisense (5′-GATCTCTAGATTACTTGTACAGCTCGTCCATGC-3′) primers, which include additional *XhoI* and *XbaI* sites (underlined), to enable directional cloning of the GFP fragment. The cDNAs encoding the modified tethered ligand and the GFP PCR product were then digested (*Xho*I/*Xba*I) and ligated. The composition of the resulting GFP-labeled tethered ligand constructs is shown in Figure S1A. The cDNAs encoding the GIP-R with monomeric cherry fluorescent protein (CHE) at the C-terminus were generated using a parallel strategy. Briefly, the stop codon in the receptor cDNA was replaced by a *XhoI* site using site-specific mutagenesis. The CHE coding sequence was amplified by PCR (using the same primers specified above), digested with *Xho*I and *Xba*I, and then ligated at the 3′ end of the receptor-coding sequence. The reporter plasmid including CHE under the control of a multimerized cAMP-responsive element (CRE_6x_-CHE) was produced by modification of a previously described CRE_6x_-luciferase (LUC) construct [15]. Using this template, the LUC coding sequence was substituted by that of CHE. The nucleotide sequence of all tethered ligands, receptor and reporter constructs was confirmed by automated DNA sequencing.

### Synthesis of [G^7^]GIP and EXE4-GM derivatives

The peptides GIP and [G^7^]GIP were synthesized at the Tufts University Core Facility using solid phase peptide synthesis on ABI 431 instruments employing Fmoc chemistry. Peptides were purified by reverse HPLC (C18 columns). Synthesis of the EXE4-GM compound was performed by New England Peptide (Gardner, MA). All other ligands used in the present study were from American Peptide Company Inc. (Sunnyvale, CA).

### Cell Culture

Human embryonic kidney (HEK) 293 cells [15] were grown in Dulbecco's modified Eagle's medium (Gibco, Carlsbad, CA) supplemented with 10% fetal bovine serum, 100 U/ml penicillin G and 100 µg/ml streptomycin. The cells were maintained at 37°C in a humidified environment containing 5% CO_2_.

### Luciferase Reporter Gene Assay

Receptor-mediated signaling was assessed using a previously described luciferase assay [15], [18]. In brief, HEK293 cells were plated at a density of 3000–6000 cells per well into clear-bottom, white 96-well plates and grown for 1–2 days to ∼80% confluency. Cells were then transiently transfected using Lipofectamine^R^ reagent (Invitrogen, Carlsbad, CA) with cDNAs encoding (i) a GPCR (or the empty expression vector), (ii) a tethered ligand (where applicable), (iii) the CRE_6X_-LUC reporter gene and (iv) β-galactosidase (as a control for transfection efficiency). For experiments investigating the agonist function of soluble peptides, tethered ligand cDNA was not included in the transfection reaction. Twenty four hours after transfection, cells were incubated with or without selected soluble peptide in serum-free medium for 6 hours. Following ligand stimulation, the medium was gently aspirated, the cells were lysed and luciferase activity was quantified using a TopCount NTX after addition of Steadylite^R^ reagent (PerkinElmer, Boston, MA). A β-galactosidase assay was then performed after adding the enzyme substrate 2-Nitrophenyl β-D-galactopyranoside, and incubating at 37°C for 30–60 minutes. Substrate cleavage (an index of β-galactosidase expression was quantified by measurement of optical density at 420 nm using a SpectraMax^R^ microplate reader (Molecular Devices, Sunnyvale, CA). Corresponding values were used to normalize the luciferase data for transfection efficiency.

### Washout Experiments

The persistence of agonist activity was assessed using a luciferase-based assay adapted from a previously reported procedure [22]. Briefly, HEK293 cells were plated and transfected as described above, with the exception that 96-well plates were pretreated with poly-L-lysine to maximize cell adhesion. Twenty-four hours after transfection, cells were treated with increasing concentrations of an agonist and further incubated for 15 minutes at 37°C. Selected wells were then washed three times with serum-free medium and plates were incubated for an additional 4 hour period. Receptor-mediated activity was quantified as described.

### Assessment of Tethered Ligand and GPCR Expression Using ELISA

The surface expression levels of myc-tagged tethered ligands or HA-tagged GIP-Rs were assessed using a previously-established procedure [15], [18]. HEK293 cells grown in 96-well clear Primaria plates (BD Biosciences, Bedford, MA) were transiently transfected with either pcDNA1.1 or a cDNA encoding the relevant epitope-tagged proteins (tethered ligand and/or GIP-R). In selected experiments, 24 hours post-transfection, cells were treated with soluble ligands and incubated for an additional 24 hour period. Forty-eight hours post-transfection, the cells were washed once with phosphate buffered saline (PBS) (pH 7.4) and fixed with 4% paraformaldehyde in PBS for 10 min at room temperature. After washing with 100 mM glycine in PBS, the cells were incubated for 30 min in blocking solution (PBS containing 20% bovine serum). To detect epitope on the recombinant proteins (myc-tagged MTLs or HA-GIPR), a horseradish peroxidase (HRP)-conjugated antibody directed against the myc-tag (polyclonal, 1∶1500 in blocking buffer, cat. #ab19312, Abcam Inc) or HA-tag (monoclonal, 1∶500 in blocking buffer, clone 3F10, Roche Inc.) was then added to the cells. After 1 hour, the cells were washed five times with PBS. Fifty µl per well of a solution containing the peroxidase substrate BM-blue (3.3′-5, 5′-tetramethylbenzidine, Roche Applied Science, Indianapolis, IN) was then added. After incubation for 30 min at room temperature, conversion of this substrate by antibody-linked HRP was terminated by adding 2 M sulfuric acid (50 µl per well). Light absorbance at 450 nm was quantified as a measure of protein expression) using a SpectraMax^R^ microplate reader.

### Confocal Microscopy

HEK293 cells were plated at a density of 150,000 cells per dish onto poly-L-lysine coated 35 mm glass bottom dishes (MatTek Corporation, Ashland, MA) and grown for 1 day to ∼60–80% confluency. Cells were then transfected with cDNA encoding GIPR-CHE and a GFP-tagged tethered ligand construct. In some experiments, soluble peptides were added 24 hours after transfection and cells were incubated at 37°C for an additional 18–24 hours before imaging. Forty eight hours following transfection, the cells were washed once with PBS and fixed with 4% paraformaldehyde in PBS for 10 minutes. After washing with 100 mM glycine in PBS, cells were washed twice with PBS and subsequently kept in the same solution. Images were obtained using confocal miscroscopy (Leica TCS SP2 instrument).

### Data Analysis

Sigmoidal curve fitting of ligand concentration-response curves was done using GraphPad (GraphPad Prism software version 5.0, San Diego, CA). The same software package was used for calculating the half maximal effective concentrations (EC_50_ values), an index of ligand potency.

## Supporting Information

Figure S1 **Cartoon illustrating the use of fluorescent proteins to detect MTLs, GPCRs and reporter gene activation.** (A) Protein domains encoded by the GFP-tagged tethered ligand constructs. Amino acids are indicated by the single-letter code. (B) A schematic representation of a GFP-labeled tethered ligand interacting with a CHE-tagged GPCR. (C) Receptor-mediated signaling induced by a GFP-tagged tethered ligand leads to intracellular accumulation of CHE following activation of the CRE_6X_-CHE reporter gene.
(TIFF)

Figure S2 **Agonist-induced translocation of the GIP-R to endosomal compartments.** The impact of soluble GIP on the subcellular distribution of GIPR-CHE was explored using confocal microscopy. HEK293 cells were transiently transfected with a plasmid encoding the GIPR-CHE and a GFP-tagged version of the the endosomal marker Rab7. Twenty-four hours later, the cells were treated for 18 h with media containing 100 nM of GIP or the corresponding vehicle. The subcellular distribution of receptor was then visualized. Soluble GIP triggered internalization of the GIPR-CHE to a vesicular endosomal compartment containing the Rab7-GFP marker, as suggested by the co-localization of the corresponding fluorescent tags.
(TIFF)

Figure S3 **Introduction of G^7^ in membrane-tethered forms of VIP or PACAP27 markedly enhances receptor-mediated signaling.** Introduction of the G^7^ substitution into tVIP (A) or tPACAP27 (B) markedly enhanced the ability of both ligands to trigger endogenous VPAC-1R -mediated signaling. HEK293 cells were transiently transfected with cDNAs encoding a tethered ligand and a CRE_6X_-LUC reporter gene construct. Twenty four hours post-transfection, ligand-induced activity was quantified. All activity data were normalized relative to the corresponding wild-type tethered VIP or PACAP construct, as indicated. (C) Sequence comparison of human GIP, VIP, PACAP27, GLP-1 and EXE4 hormones. Position 1 represents the N-terminal residue of the peptides. A highly conserved helix-capping motif among class B1 hormones includes residue 7 (red), as well as positions 6 and 10 (blue) (Neumann et al. 2008; Parthier et al. 2009). This sequence motif is identical between GIP, VIP and PACAP27. Data represent the mean ± SEM from at least 3 independent experiments, each performed in quadruplicate.
(TIFF)
