# Supplementary figures and images for: Discovery of Dual-Action Membrane-Anchored Modulators of Incretin Receptors
Source: PLoS One. 2011 Sep 14;6(9):e24693. doi: 10.1371/journal.pone.0024693 (PMC3173463; doi:10.1371/journal.pone.0024693)

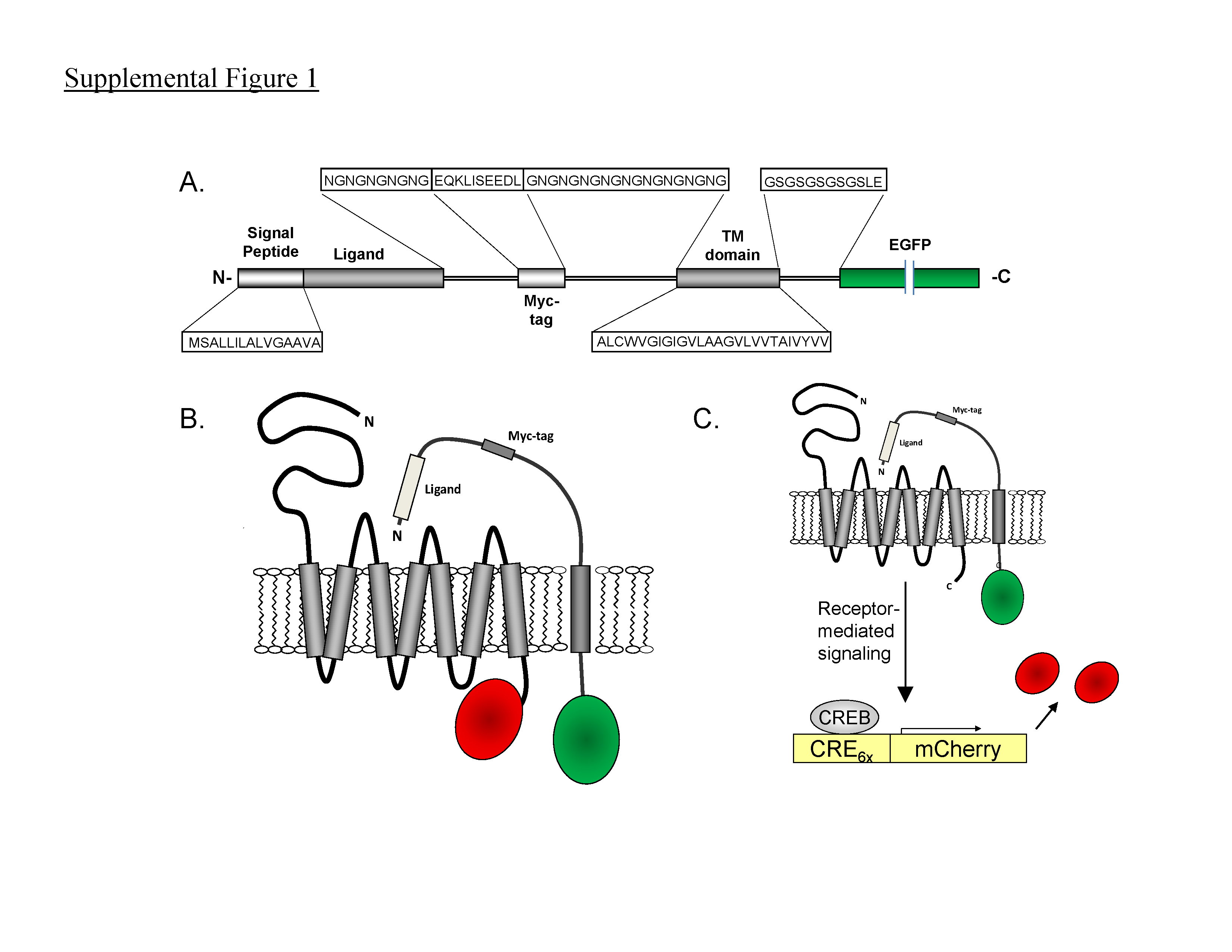

Supplement: Figure S1 — Cartoon illustrating the use of fluorescent proteins to detect MTLs, GPCRs and reporter gene activation. (A) Protein domains encoded by the GFP-tagged tethered ligand constructs. Amino acids are indicated by the single-letter code. (B) A schematic representation of a GFP-labeled tethered ligand interacting with a CHE-tagged GPCR. (C) Receptor-mediated signaling induced by a GFP-tagged tethered ligand leads to intracellular accumulation of CHE following activation of the CRE6X-CHE reporter gene. (TIFF) [file pone.0024693.s001.tiff]

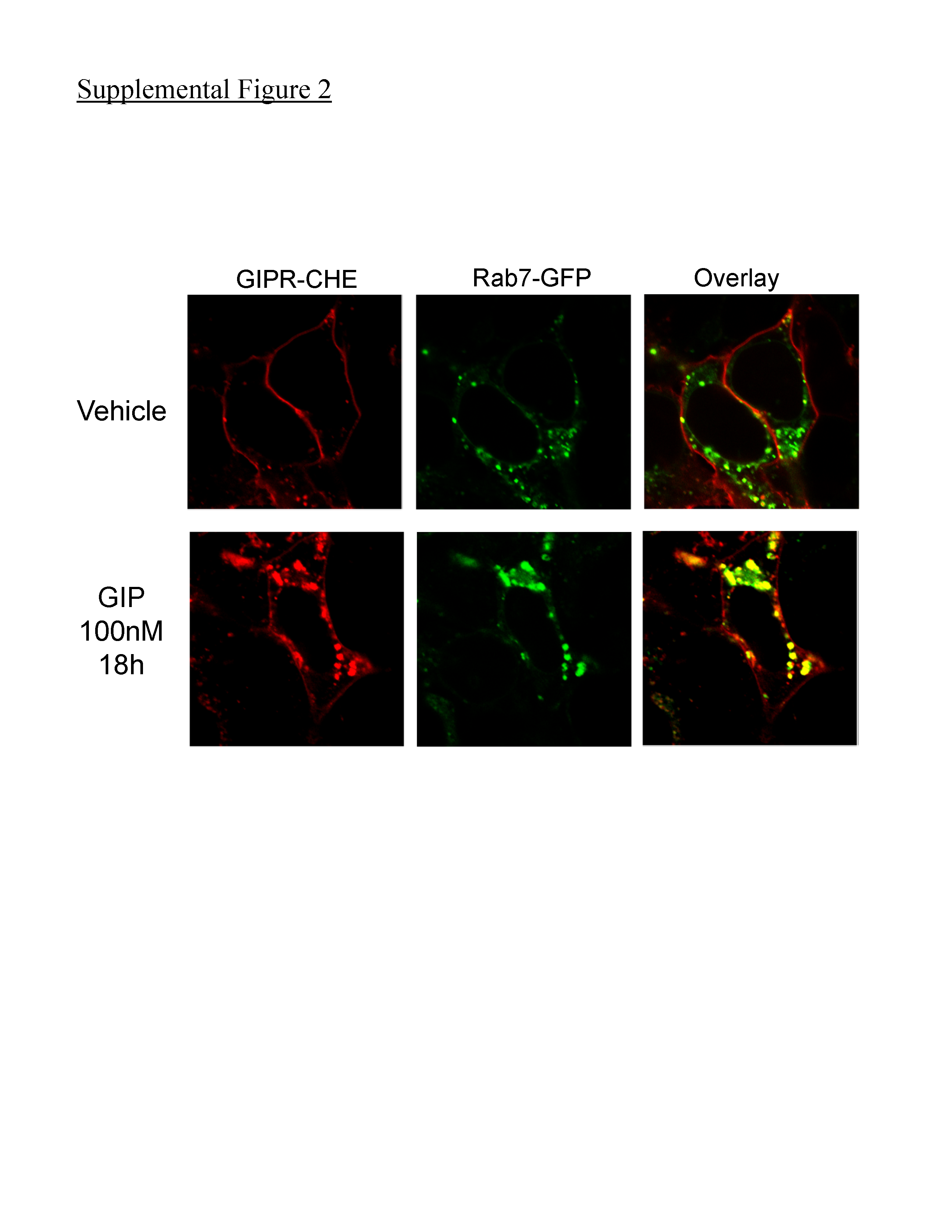

Supplement: Figure S2 — Agonist-induced translocation of the GIP-R to endosomal compartments. The impact of soluble GIP on the subcellular distribution of GIPR-CHE was explored using confocal microscopy. HEK293 cells were transiently transfected with a plasmid encoding the GIPR-CHE and a GFP-tagged version of the the endosomal marker Rab7. Twenty-four hours later, the cells were treated for 18 h with media containing 100 nM of GIP or the corresponding vehicle. The subcellular distribution of receptor was then visualized. Soluble GIP triggered internalization of the GIPR-CHE to a vesicular endosomal compartment containing the Rab7-GFP marker, as suggested by the co-localization of the corresponding fluorescent tags. (TIFF) [file pone.0024693.s002.tiff]

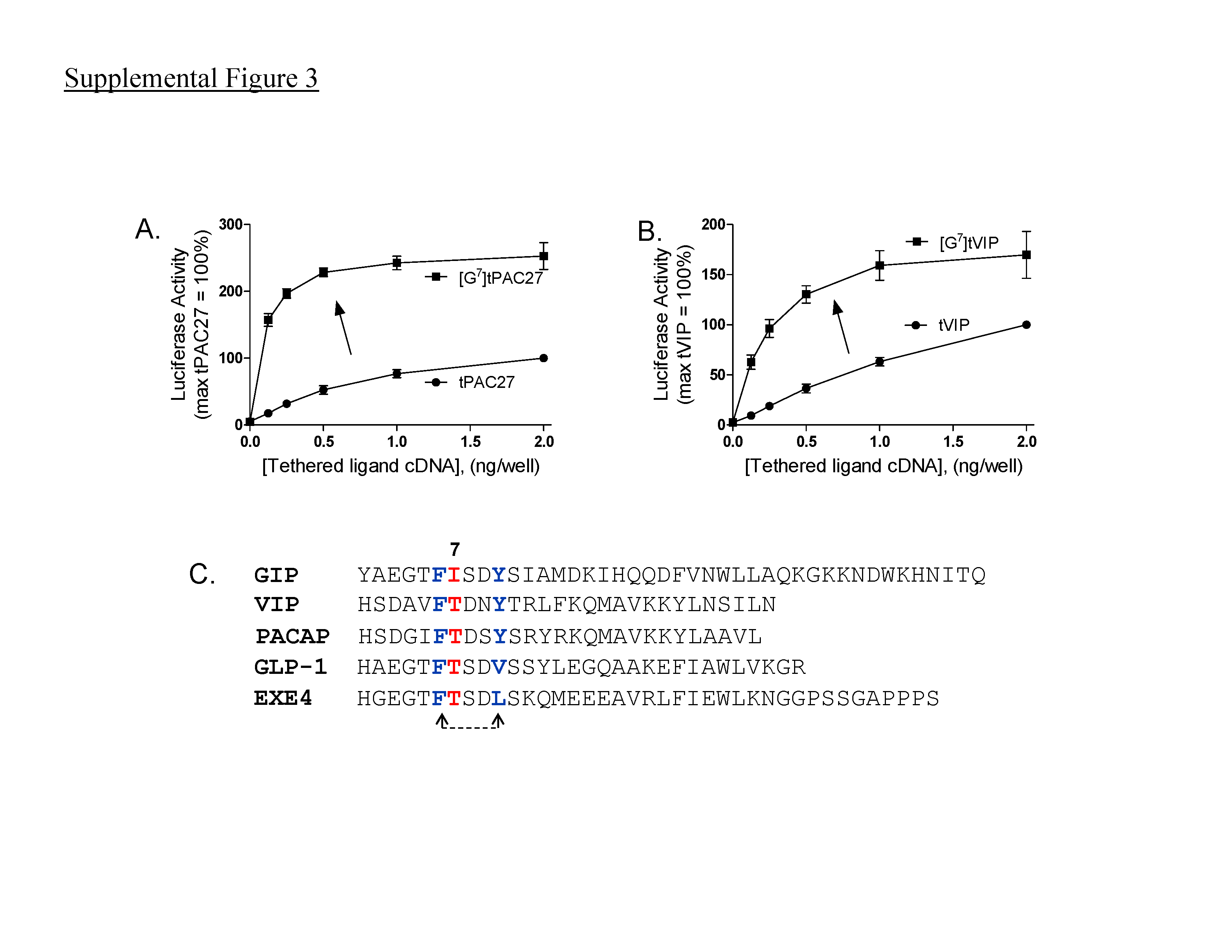

Supplement: Figure S3 — Introduction of G7 in membrane-tethered forms of VIP or PACAP27 markedly enhances receptor-mediated signaling. Introduction of the G7 substitution into tVIP (A) or tPACAP27 (B) markedly enhanced the ability of both ligands to trigger endogenous VPAC-1R -mediated signaling. HEK293 cells were transiently transfected with cDNAs encoding a tethered ligand and a CRE6X-LUC reporter gene construct. Twenty four hours post-transfection, ligand-induced activity was quantified. All activity data were normalized relative to the corresponding wild-type tethered VIP or PACAP construct, as indicated. (C) Sequence comparison of human GIP, VIP, PACAP27, GLP-1 and EXE4 hormones. Position 1 represents the N-terminal residue of the peptides. A highly conserved helix-capping motif among class B1 hormones includes residue 7 (red), as well as positions 6 and 10 (blue) (Neumann et al. 2008; Parthier et al. 2009). This sequence motif is identical between GIP, VIP and PACAP27. Data represent the mean ± SEM from at least 3 independent experiments, each performed in quadruplicate. (TIFF) [file pone.0024693.s003.tiff]
